# The Exposome and Immune Health in Times of the COVID-19 Pandemic

**DOI:** 10.3390/nu14010024

**Published:** 2021-12-22

**Authors:** Javier S. Morales, Pedro L. Valenzuela, Adrián Castillo-García, Javier Butragueño, David Jiménez-Pavón, Pedro Carrera-Bastos, Alejandro Lucia

**Affiliations:** 1 MOVE-IT Research Group, Department of Physical Education, Faculty of Education Sciences, Universidad de Cádiz, 11519 Cadiz, Spain; david.jimenez@uca.es; 2 Biomedical Research and Innovation Institute of Cádiz (INiBICA) Research Unit, Puerta del Mar University Hospital, University of Cádiz, 11009 Cadiz, Spain; 3 Faculty of Sport Sciences, Universidad Europea de Madrid, 28670 Madrid, Spain; pedroluis.valenzuela@universidadeuropea.es (P.L.V.); alejandro.lucia@universidadeuropea.es (A.L.); 4 Physical Activity and Health Research Group (‘PaHerg’), Research Institute of the Hospital 12 de Octubre (‘imas12′), 28041 Madrid, Spain; 5 Fissac—Physiology, Health and Physical Activity, 28015 Madrid, Spain; adriancastillogarcia@icloud.com; 6 LFE Research Group, Department of Health and Human Performance, Faculty of Physical Activity and Sport Sciences, Polytechnic University of Madrid (UPM), 28040 Madrid, Spain; javier.butragueno@gmail.com; 7 CIBER of Frailty and Healthy Aging (CIBERFES), 28029 Madrid, Spain; 8 Centre for Primary Health Care Research, Lund University, Skane University Hospital, 205 02 Malmö, Sweden; pmcbastos@gmail.com; 9 Faculty of Biomedical and Health Sciences, Universidad Europea de Madrid, 28670 Madrid, Spain

**Keywords:** healthy lifestyle, environmental exposure, COVID-19, vaccines, infectious diseases

## Abstract

Growing evidence supports the importance of lifestyle and environmental exposures—collectively referred to as the ‘exposome’—for ensuring immune health. In this narrative review, we summarize and discuss the effects of the different exposome components (physical activity, body weight management, diet, sun exposure, stress, sleep and circadian rhythms, pollution, smoking, and gut microbiome) on immune function and inflammation, particularly in the context of the current coronavirus disease 2019 (COVID-19) pandemic. We highlight the potential role of ‘exposome improvements’ in the prevention—or amelioration, once established—of this disease as well as their effect on the response to vaccination. In light of the existing evidence, the promotion of a healthy exposome should be a cornerstone in the prevention and management of the COVID-19 pandemic and other eventual pandemics.

## 1. Introduction

After the reemergence of the Severe Acute Respiratory Syndrome (SARS) Coronavirus (SARS-CoV) in 2003, Cheng et al. [1] warned (in 2007) of the need to take measures aimed at preventing the possibility that SARS—or other viruses—returning if conditions are fit for their introduction, mutation, amplification, and transmission. In light of the more than 233 million cases and 4.7 million deaths worldwide, according to data from The Johns Hopkins University (updated as of 30 September 2021) [2], caused by the current pandemic of coronavirus disease 2019 (COVID-19), it seems clear that countries are not prepared to deal with an emergency that had already been predicted 13 years earlier. Yet, the COVID-19 pandemic is just one of the many pandemics that are likely to come in the foreseeable future [3].

**Figure 1:**
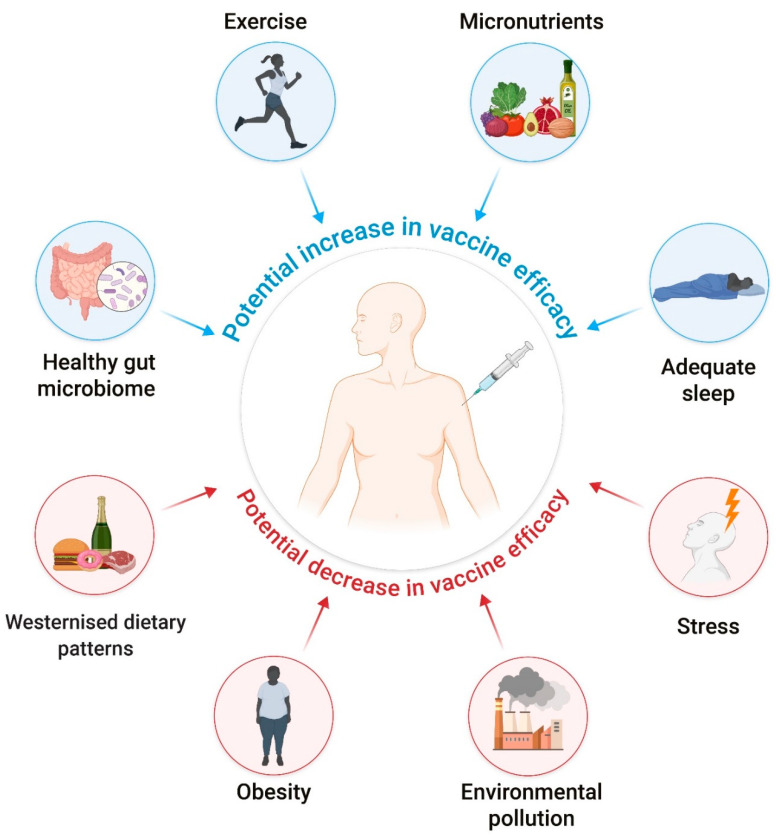
Summary of potential mechanisms underlying the positive and negative effects of the different exposome components on vaccine efficacy. Source: Self-elaboration based on the main results obtained in the scientific literature.

Combining a healthy lifestyle with environmental exposure could be an important companion measure to vaccines and medications for the prophylaxis and treatment of future pandemics (Figure 1). The implementation of healthy lifestyles and environmental exposures can still play a key role in the context of the current COVID-19 pandemic, potentially contributing to the prevention of new cases or the improvement of the prognosis of infected patients [4,5,6,7,8]. These lifestyle measures will be explained in detail from Section 2 to Section 9 and mainly include performing regular physical activity, avoiding obesity, following a diet rich in fresh fruits, vegetables, polyphenols, micronutrients and fish-derived omega-3 fatty acids (e.g., the Mediterranean diet) that together can contribute to attenuate inflammation, minimizing psychosocial stress and exposure to environmental pollutants, following healthy sleeping patterns, and avoiding smoking. In this regard, a study conducted with twins revealed that, compared with genetic endowment, non-heritable factors seem to be the strongest contributors to individual variability in immune responses [9]. Indeed, various lifestyles and environmental factors cannot only modulate immune responses [10,11,12,13] but also individual response to vaccination [14].

The relevance of lifestyle/environmental factors for health has been extensively evidenced [15]. However, most epidemiological studies have focused almost exclusively on single exposures. Since health status obviously depends on multiple variables [16], a more comprehensive paradigm that considers the exposure to different endogenous and environmental factors collectively is emerging, the so-called exposome. Briefly, the exposome refers to life-course exposures starting from the prenatal period onward [17]. This holistic approach might help to gain insight into the influence of lifestyle/environmental factors on human health [18,19,20,21,22]. The exposome encompasses two broad categories of non-genetic exposures: individual-level (physical activity, weight management, diet, stress, sleep and circadian rhythms, pollution, smoking, as well as the gut microbiome) and general exposures (climate and sunlight, environmental pollution), respectively [23]. Of interest, all these exposures can act synergistically through common mechanisms, such as the nuclear factor binding near the k light-chain gene in the B cells (NF-κB) family of transcriptional factors and the inflammasome machinery—the innate immune system receptors and sensors that regulate the activation of caspase-1 and induce inflammation in response to infectious microbes and host-derived molecules, the so-called damage-associated molecular patterns—and subsequent inflammation [24].

In the present narrative (non-systematic) review, we aim to summarize the effect of the exposome (including physical activity, weight management, diet, vitamin D and sun exposure, stress, sleep and circadian rhythms, exposure to environmental pollution, smoking, and gut microbiome) on inflammation and immune function, with a focus on the potential role of lifestyle changes in the prevention—or amelioration once established—of infectious diseases such as COVID-19, as well as their influence on the efficacy of vaccines. Our objective is to draw attention to the potential importance of complementary lifestyle-related measures in order to deal with the COVID-19 pandemic and possible future pandemics, which should be implemented along with other established measures such as vaccination and medical treatments.

## 2. Physical Activity

Approximately one-quarter of the population worldwide is considered physically inactive (that is, not meeting the minimum international recommendations, i.e., at least 150 or 75 min/week of moderate (such as walking) or vigorous (such as very brisk walking) aerobic activities, respectively) [25]. There is evidence that in the context of the current pandemic, preventive measures such as social distancing and forced lockdown have increased sedentary behaviors and physical inactivity [26,27,28,29,30]. Of relevance, physical inactivity can promote baseline inflammation and several related pathophysiologic alterations including, among others, insulin resistance, dyslipidemia, vascular endothelial dysfunction, high blood pressure, and sarcopenia [24,31]. Consistent with these effects, physical inactivity has been described as a contributor to over 35 chronic conditions [31].

Conversely, the beneficial effects of regular physical activity (PA) on immune function are well documented. Regular PA is associated with a 31% and 37% risk reduction of community-acquired infectious diseases and subsequent mortality, respectively, compared to inactive controls [32]. Physical exercise interventions can increase CD4 lymphocyte counts and salivary immunoglobulin A (IgA) concentration and decrease neutrophil counts compared to controls [32]. In fact, even just four weeks of either moderate- or high-intensity interval exercise can lead to a remarkable improvement in natural killer (NK) cell number and function (i.e., ‘killing capacity’) [33]. Regular exercise can also attenuate *immunosenescence* [34], that is, the progressive immune dysfunction that occurs as we age, with remodeling of lymphoid organs and a higher susceptibility to infections.

Acute bouts of exercise also provide some benefits to the immune system, stimulating the interchange of innate immune cells between lymphoid tissues and the blood compartment, while improving immunosurveillance against pathogens and decreasing systemic inflammation [35]. Indeed, muscle contractions induce the release of hundreds of molecules—mostly but not only small peptides (cytokines) collectively known as *myokines*—from skeletal muscles (but also from other tissues, in which case they are broadly termed *exerkines*) into the bloodstream, thereby reaching other tissues and organs where they elicit myriad beneficial effects, including anti-inflammatory ones [36,37]. Thus, the beneficial effects of regular PA can be attributed, at least in part, to the accumulation of frequent, repeated ‘time windows’ (i.e., during exertion and in the following hours) where myokines (or *exerkines*) are being released to the blood with the subsequent salutary effects. Notably, when it is released from the working muscles and thus acts as a myokine, interleukin (IL)-6 exerts anti-inflammatory effects and can stimulate NK cells, an effect that is not observed under resting conditions. In fact, IL-6 released from other sources, such as immune cells, in a non-exercise milieu, has a pro-inflammatory role [38].

A high level of cardiorespiratory fitness (CRF), which can be achieved through regular exercise practice, has been associated with fewer days (−46%) of illness from acute respiratory infections as compared with a low level of CRF [39]. A high CRF has also been reported to positively impact the expression of immune markers that could theoretically reduce the risk of COVID-19 complications [40], particularly the so-called *cytokine storm* syndrome [40]; that is, the excessive, uncontrolled release of proinflammatory cytokines (e.g., interferon-γ, IL-1, IL-6, IL-18, tumor necrosis factor [TNF]-α) to the bloodstream that is frequently found in patients with severe disease, including COVID-19 [41]. In the same line, CRF has been reported to be independently and inversely associated with the likelihood of hospitalization for COVID-19 [42].

Physical exercise might also be beneficial in improving the efficacy of vaccines against SARS-coronavirus (CoV)-2 [32,43] and other infectious agents. Both ‘acute’ (i.e., a single session) or regular exercise (repeated sessions) prior to influenza vaccination are safe and can enhance the immune response to vaccination [44,45]. Edwards et al. showed that performing eccentric contractions of the deltoid and biceps brachii muscles of the nondominant arm 6 h before influenza vaccination in the same arm improved cell-mediated response (as reflected by enhanced interferon-γ responses) in men and increased antibody responses in women [46]. A meta-analysis by Chastin et al. found that regular exercise increases antibody titers after vaccination against influenza, pneumococcal, or varicella zoster virus, respectively [32]. There is also evidence from interventional research supporting the beneficial role of regular exercise. Notably, a study with participants aged ~70 years who were previously sedentary and had poor influenza vaccine responses found that those randomized to moderate-intensity cardiovascular exercise showed marked improvements in influenza seroprotection throughout the entire influenza season compared to the control group [47]. In addition, exercise may minimize the deleterious effects of immunosenescence on vaccination efficacy by maintaining the peripheral T-cell pool and the ability of these cells to respond to novel vaccine antigens. Physically active, older adults are known to have fewer and more ‘senescent’ and naïve T cells, respectively, than their sedentary counterparts. Importantly, preserving a diverse pool of both functional (non-senescent) and naïve T-cells is likely to reduce infection risk, and the regular release of muscle-derived cytokines such as IL-7 and IL-15 has been purported to play important roles in the beneficial effects of exercise on immunity [34]. Furthermore, elderly women who were physically active had a better immune response after vaccination than those who were less active [48]. Although more studies are needed to confirm its efficacy, acute exertion (i.e., a single session of relatively intense exercise performed just prior to vaccination) has also been postulated as an effective strategy to increase the immune response to vaccination [44].

## 3. Body Weight Management

The worldwide prevalence of obesity has almost tripled since 1975, with 39% and 13% of adults now considered to have overweight and obesity, respectively [49]. Excessive adiposity, especially central adiposity—accumulation of fat in the lower torso around the abdominal area—is detrimental to health, with consistent evidence showing that overweight and obesity are associated with an increased risk of associated comorbidities—mainly, but not only, cardiovascular disease (CVD) [50]. Furthermore, obesity is overall associated with accelerated ageing and subsequent immune dysfunction, the so-called *adipaging* [51].

There is meta-analytical evidence that individuals with obesity are not only at greater risk of COVID-19 infection but also of having a worse prognosis (higher risk of severe disease and mortality) than their normal-weight peers [52,53,54]. Several mechanisms could contribute to the detrimental effects of obesity on immune function. Excess of adiposity, particularly around abdominal organs (i.e., visceral adipose tissue), is characterized by increased production and secretion of pro-inflammatory cytokines and other molecules, the so-called *adipocytokines* or *adipokines* [55], which could lead to low-grade chronic inflammation (LGCI) and contribute to several chronic inflammatory conditions. Excess of adiposity has been reported to exert modulatory effects on key populations of immune cells that are critical for ensuring an adequate response to SARS-CoV-2 [56]. Obesity is also associated with both a reduced number of NK lymphocytes and a lower cytotoxic capacity of these cells [57,58]. On the other hand, leptin, one of the most abundant adipokines produced by adipocytes, affects both innate and adaptive immunity [59,60]. Notably, leptin increases the production of pro-inflammatory cytokines in monocytes and macrophages [61]. Thus, a positive correlation between circulating leptin and inflammatory biomarkers (C-reactive protein [CRP], IL-6, TNF-α) has been suggested [62]. In this context, obesity is frequently associated with leptin resistance, a phenomenon traditionally attributed to impaired transport of this molecule through the blood–brain barrier that leads to increased leptin levels [63]. This, in turn, leads to the dysregulation of cytokine production, increased susceptibility to infectious diseases, autoimmune conditions, and upregulated inflammatory responses [60], and could explain why many obesity-associated comorbidities have been linked to immune dysfunction [64]. Moreover, angiotensin converting enzyme (ACE) 2 expression in adipose tissue exceeds that of the lung tissue [65]. Since ACE2 is an important entry receptor for SARS-CoV-2 [66], elevated levels of this membrane receptor, as a consequence of excess adipose tissue, could promote viral entrance into target cells and increase the risk of COVID-19 infection.

Some comorbidities linked to obesity are also associated with higher COVID-19 severity. Obesity is often associated with respiratory dysfunction, which increases the risk of hypoventilation, pulmonary hypertension, and cardiac stress, worsening COVID-19 prognosis [67]. People with obesity have impaired ventilatory mechanics, as excess adiposity causes a decrease in expiratory reserve volume, leading to lower levels of both functional residual capacity and total lung capacity [68]. Overall, these effects can lead to lower CRF levels which, as mentioned above, have been inversely associated with the risk of COVID-19 hospitalization [42].

Obese individuals show an impaired response to vaccination compared to normal-weight individuals, as a recent study with 248 healthcare workers who were vaccinated against COVID-19 with the second dose of the BNT162b2 vaccine suggests [69]. This is in line with previous evidence that obesity could decrease the immune response after vaccination against hepatitis B [70,71], rabies [72], tetanus [73], or influenza [74]. Different mechanisms might explain an obesity-induced impairment in response to vaccination. On the one hand, due to excessive adiposity, individuals with obesity could receive a lower relative vaccination dose or experience reduced absorption from the site of injection [73]. Alternatively, obesity-induced LGCI might reduce the immune response to vaccines [73].

In summary, excess adiposity can lead to a pro-inflammatory status, also known as metaflammation [75], with subsequent immune dysfunction (e.g., impaired innate and adaptative response to infectious agents and vaccines) and respiratory dysfunction, thereby worsening prognosis after virus infections. Thus, body weight management should be a key public health concern in the prevention/management of the current COVID-19 pandemic and future pandemics.

## 4. Diet

**Figure 2:**
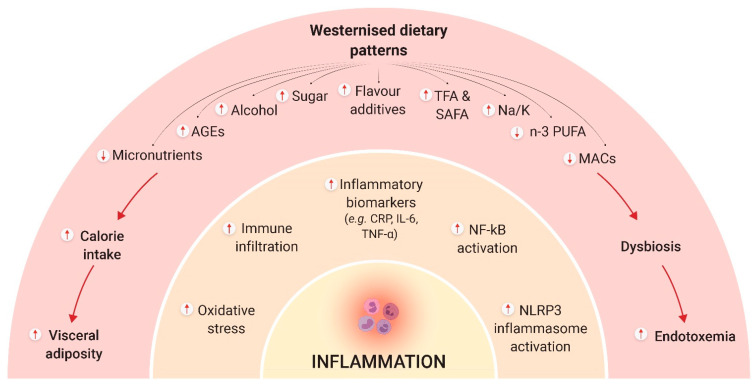
Summary of potential mechanisms underlying the deleterious effects of westernised dietary patterns on inflammation. Abbreviations: AGEs, advanced glycation end products; CRP, C-reactive protein; IL-6, interleukin-6; MACs, microbiota-accessible carbohydrates; NF-κB, nuclear factor-κB; PUFA, polyunsaturated fatty acids; SAFA, saturated fatty acids; TFA, trans fatty acids; TNF, tumor necrosis factor. Source: Self-elaboration based on the main results obtained in the scientific literature.

The westernised way of life has brought several changes to the human diet, particularly the widespread consumption of ultra-processed foods with a high content of fat, sugar, salt, and flavour additives that can cause an excess calorie intake [76]. These features contribute to obesity, which, as previously discussed, can lead to a proinflammatory status, impair immune function, and increase the risk of many chronic diseases (Figure 2).

The abundance of food and the way it is consumed in Western countries has led to dietary patterns characterized by several meals per day consumed in a very long eating window. Thus, feeding periods longer than 14 h have been described in overweight individuals [77]. However, when overweight individuals with >14 h eating duration ate for only 10–11 h per day over 16 weeks, they reduced their energy intake by 20% and showed a reduction in total body weight (−3.27 kg) and body mass index (−1.15 kg/m^2^) [77]. In this effect, intermittent fasting and particularly time-restricted feeding (TRF) protocols have gained popularity in recent years because they might help adults with obesity to lose weight. However, controversy exists, and in fact, meta-analytical evidence indicates no significant difference in weight loss when comparing intermittent (i.e., TRF) or continuous energy restriction interventions [78,79,80,81]. This being said, TRF might produce larger metabolic benefits even in the absence of weight loss, including increases in insulin sensitivity and decreases in blood pressure or oxidative stress [82].

Controversy also exists on the influence of TRF on inflammation. The available evidence suggests that TRF has no effects on inflammatory markers such as CRP, IL-6, or TNF-α [82,83,84,85,86], although it is possible that different types of TRF could produce different effects. For instance, a study conducted during the Ramadan period, 16 h fasting from sunrise to sunset throughout 29 consecutive days, showed that this type of TRF increased IL-6 levels but also induced a reduction in TNF-α and CRP [87]. Other beneficial effects of intermittent fasting could involve metabolic switching and cellular resistance stress, although the specific mechanisms remain unclear [88].

Beyond its obesogenic effect, westernized diets represent a major source of advanced glycation end products (AGEs) [89]; that is, proteins, nucleic acids, or lipids that become non-enzymatically glycated as a result of exposure to reducing sugars. Dietary AGEs are typically found in foods cooked under dry heat (such as grilling, broiling, roasting, and frying) or exposed to thermal treatments, with processed and ultra-processed foods being a major source of these compounds. A systematic review of randomized controlled trials (RCT) found that AGEs-rich diets can increase TNF-α as well as 8-isoprostane, a marker of oxidative stress [90]. There is a growing body of evidence that closely links AGEs with chronic diseases [89,91,92]. Conversely, meta-analytical evidence suggests that reducing dietary AGEs can lower circulating AGEs as well as the receptor for these compounds, which translates into a reduction in TNF-α, vascular cell adhesion molecule-1 (VCAM-1), 8-isoprostane, and leptin, respectively, together with an increase in adiponectin and sirtuin-1 [93]. Western diets typically also include high glycemic load ingredients, such as sugars and refined cereal grains. These have been shown, in mononuclear cells from healthy individuals, to increase the generation of reactive oxygen species (ROS) and activate redox-sensitive transcription factors, such as NF-κB, which upregulates the expression of various proinflammatory genes [94,95]. Other potential dietary triggers of inflammation that are present in excess in the Western diet include alcohol, salt, industrial trans fatty acids, and certain saturated fatty acids (particularly palmitic acid), all of which have been shown to cause inflammation through different mechanisms [96,97,98,99]. For instance, a high-salt diet may favour polarization of macrophages towards a pro-inflammatory (M1) phenotype, skew the balance between the proinflammatory T helper (Th)17 lymphocytes and the anti-inflammatory T regulatory (reg) cells, and may adversely change the microbiome [96], which, as will be discussed later, also affects inflammation and overall immune function. Conversely, increasing potassium intake has been shown to neutralize salt-loading-induced Th17 activation [100,101].

Giving support to the role of dietary factors in LGCI, various intervention studies have shown that hypercaloric fast-food meals (which are high in AGEs, sugars, refined grains, salt, and hydrogenated or saturated fats) increase the concentration of various proinflammatory molecules, even in lean healthy individuals [102,103]. Moreover, there is evidence associating westernised diets with increased serum CRP levels [104]. Westernised dietary patterns also tend to be low in fresh fruits and vegetables. This is relevant since a low intake of these food groups is considered one of the main diet-related risk factors according to the Global Burden of Disease Study [105,106] that estimated that poor dietary patterns were responsible for 11 million deaths worldwide (even outrunning smoking) in 2017 [105]. Interestingly, since fruits and vegetables are major sources of the so-called microbiota-accessible carbohydrates, reducing their intake can decrease the richness and diversity of the gut microbiota, which, as reviewed below, can contribute to LGCI [107,108]. Conversely, diets with a high content of fruits and vegetables have been reported to decrease circulating concentrations of TNF-α and CRP while increasing gamma-delta T lymphocytes (a group of T cells that are abundant in the gut mucosa known as ‘intraepithelial lymphocytes’) [109]. Foods that are rich in fruits or vegetables have also been shown to prevent or attenuate the oxidative and inflammatory stress induced by hypercaloric fast food meals [110,111,112,113]. This has mainly been attributed to their high content of several bioactive compounds involved in the regulation of genes that affect the inflammatory response and antioxidant status [114,115]. In addition, foods rich in fruits and vegetables are high in various minerals and vitamins reported to decrease oxidative stress and inflammation while improving overall immune function (i.e., potassium [100,101], magnesium [116,117], and vitamins B9 (folate [118,119,120]), C [121,122], and E [123]).

Other important micronutrients for immune health are zinc, copper, iron, and selenium, as well as vitamins A, B6, B12, and D. The impact of these micronutrients on viral infections has been extensively reviewed elsewhere [5,120,124,125]. As for vitamin D, it will be discussed at length in a later section. Of note, the nutritional status of zinc, copper, and selenium has been correlated with severity and mortality due to COVID-19 [126,127,128]. Accordingly, two observational studies found an association between zinc supplementation and better outcomes in COVID-19 hospitalized patients [129,130]. However, supplementation with high-dose zinc, vitamin C, or a combination of the two nutrients in ambulatory COVID-19 patients did not reduce the duration of symptoms compared with standard care [131]. Patients who might benefit the most from increasing the intake of these nutrients are those who are zinc and/or vitamin C-deficient [132], or have severe and critical COVID-19 since those typically present with high-grade inflammation [133], which can significantly affect the status of zinc [134] and vitamin C [135]. What seems clear is that, in times of the pandemic, it is essential to cover any nutritional deficiencies through an adequate diet and specific nutritional supplementation if applicable [136].

An optimal nutritional status can affect not only the outcome of an infection but also vaccination efficacy. Indeed, malnutrition might produce a lower antibody response to vaccination in children [137,138,139,140,141]. On the other hand, chronic overfeeding can cause obesity, which, as mentioned above, can lead to an impaired response to vaccination [142,143]. Interestingly, not only lean malnourished individuals but also obese people can present with multiple micronutrient deficiencies [144,145,146]. This is relevant because there is some evidence (albeit still limited) that zinc, copper, selenium, and iron, as well as vitamins A, B6, B9, B12, C, D, and E, might affect the immune response to various vaccines [124].

The daily consumption of five or more portions of fruits and vegetables per day has been shown, in an RCT, to improve the antibody response to Pneumovax II vaccination in healthy participants aged 65–85 years [147]. A study that included 3,042 individuals of both sexes showed that those with higher adherence to the Mediterranean diet (characterized by a high intake of fruits, vegetables, legumes, nuts, whole grains, fish and olive oil, moderate consumption of dairy products and red wine, and low consumption of red meat [148]) had 20%, 17%, 14%, 15%, and 6% lower levels of CRP, IL-6, white blood cell counts, homocysteine, and fibrinogen, than individuals with low adherence to the diet, respectively [149]. Consuming a Mediterranean diet modulated specific components of the gut microbiome of non-frail or pre-frail participants from several European countries, with microbiome changes associated with a reduction in the risk of frailty, an improvement in cognitive function, and a decrease in the circulating levels of two inflammatory markers, high-sensitivity CRP (hsCRP) and IL-17 [150]. In addition, results from the Moli-sani study including 14,586 healthy individuals showed that white blood cell and platelet counts were both inversely related to Mediterranean diet adherence [151]. Meta-analytical evidence indicates that high adherence to a Mediterranean diet attenuates inflammation and improves vascular endothelial function by increasing adiponectin and flow-mediated dilatation while decreasing hsCRP, IL-6, and intracellular adhesion molecule-1 [152]. Accordingly, adherence to the Mediterranean diet has been associated with a lower incidence (and related mortality) of CVD, cancer, and neurodegenerative conditions, as well as with all-cause mortality [153]. Even if compared to a low-fat diet, a Mediterranean diet supplemented with extra-virgin olive oil or nuts has been associated with a lower rate of major CVD events in individuals at high risk for CVD [154]. These results might be explained by the salutary—notably, antioxidant and immunomodulator—effects of certain dietary components of the Mediterranean diet. These include polyphenols [155,156] (found in fruits and vegetables, nuts, and extra virgin olive oil), micronutrients (such as magnesium [116,117], vitamins B9 [118,119,120], C [121,122] and E [123]), and fish-derived omega-3 fatty acids (which stimulate the resolution of inflammation by giving rise to molecules, the so-called specialized proresolving mediators) [157]. Hence, due to its antioxidant, anti-inflammatory and immunomodulatory benefits, and its protective effect against predictors of morbidity and mortality in patients with COVID-19 such as CVD, the Mediterranean diet could be a promising and relatively easy-to-apply method to attenuate the severity of SARS-CoV-2 and eventual future viral pandemics [158,159]. In fact, a study showed that adherence to the Mediterranean diet was inversely associated with COVID-19 cases and related deaths in Spain and across 23 Organization for Economic Co-operation and Development countries [160].

## 5. Vitamin D and Sun Exposure

While traditionally known for its role in bone metabolism and skeletal muscle function [161], vitamin D has recently gained attention as an important player in immune health [162]. Vitamin D modulates both the adaptive and innate arms of the immune system [162] and might improve the inflammatory response to viral infection [163,164]. In fact, a Mendelian randomization study with 35,833 participants showed that low levels of plasma 25-hydroxyvitamin (OH)D, a biomarker of vitamin D status, were associated with a higher risk of bacterial pneumonias during a 38-year follow-up [165]. Additionally, vitamin D deficiency has been associated with an increase in proinflammatory cytokines (TNF-α, IL-6) [166,167]. Preclinical studies have shown that vitamin D polarizes macrophages towards an anti-inflammatory phenotype, thereby reducing the secretion of proinflammatory cytokines such as IL-6 or TNF-α [168,169]. Moreover, vitamin D can polarize T CD4+ lymphocytes from a pro- (Th1/Th17) to an anti-inflammatory (Treg) phenotype, respectively [162]. Furthermore, this vitamin regulates the expression of genes that code for antimicrobial proteins in dendritic cells and macrophages [162]. A recent systematic review and meta-analysis of case-control, cross-sectional, and prospective cohort studies showed a significant and non-linear correlation between 25(OH)D levels below 37.5 nmol/L on the one hand, and risk and severity of acute respiratory tract infection on the other [170]. There is also meta-analytical evidence that vitamin D supplementation can reduce the incidence of respiratory infections and asthma exacerbations, especially in people with vitamin D deficiency [171,172].

Hence, vitamin D can affect the prognosis of COVID-19. Those European countries with lower reported plasma levels of vitamin D in the population, such as Spain and Italy (especially in older people), had the highest mortality rates from COVID-19 early in the pandemic [173,174]. In addition, vitamin D deficiency has been associated with a greater susceptibility to COVID-19 infection [175,176] and a greater risk of intensive care unit (ICU) admission in infected patients [177], with lower vitamin D levels reported for patients with a severe course of the disease [178,179]. Accordingly, there is evidence suggesting that vitamin D supplementation can have a positive effect on COVID-19 symptoms and severity. Compared with a lower dose (1000 IU), daily oral supplementation with 5000 IU of vitamin D3 for two weeks reduced the time to recovery of symptoms such as cough and gustatory sensory loss among mild-to-moderate COVID-19 patients with sub-optimal vitamin D status [180]. Further, a meta-analysis including 13 studies and 2933 patients found that vitamin D supplementation was associated with a reduced risk of adverse outcomes, ICU admission, and mortality from COVID-19 [181]. Interestingly, vitamin D supplementation was associated with improved clinical outcomes, especially when administered after the diagnosis of COVID-19 and not in patients who received vitamin D before diagnosis [181]. The benefits of vitamin D on COVID-19 complications could be due to its effects on the production of antimicrobial and antiviral proteins, as well as on the modulation of the inflammatory response, thereby preventing (or suppressing) the cytokine storm [182].

Yet there is some controversy on the potential benefits of vitamin D. A recent retrospective study determined that 25(OH)D levels above 40 nmol/L were not able to adequately predict in-hospital mortality in patients with COVID-19 [183]. A systematic review and meta-analysis found no significant effect of vitamin-D supplementation on major health-related outcomes in COVID-19 (such as mortality, ICU admission rates or need for invasive ventilation) [184]. A multi-center, double-blind, placebo-RCT trial did not find any beneficial effect on length of hospitalization among patients with COVID-19 receiving a single high oral dose of vitamin D3 (200,000 IU). This finding could also be explained by the fact that vitamin D supplementation prevents acute respiratory infections (e.g., COVID-19) when given as low-dose daily maintenance, but not as high-dose intermittent bolus [185,186].

The potential role of vitamin D on vaccine responses seems unclear [187]. Zimmerman et al. found that low vitamin D levels at baseline were associated with higher antibody titers in response to the human papillomavirus vaccine in young male adults [188]. However, a systematic review and meta-analysis failed to find a significant association between vitamin D status and the immunogenic response to influenza vaccination, although a lower seroprotective response to vaccination with some strains of influenza was observed in patients with vitamin D deficiency [189]. A placebo, double-blind RCT found no differences of serum levels of cathelicidin antimicrobial peptide (a polypeptide that is primarily stored in the lysosomes of macrophages and polymorphonuclear leukocytes), antibody titers, and ROS production 28 days after the influenza vaccine between cholecalciferol supplementation and placebo in deficient elderly persons, despite the former increasing vitamin D levels [190]. However, the supplementation group showed a reduced Th1/Th2 ratio after vaccination (coinciding with the end of the 3-month period with vitamin D supplementation) as well as low plasma levels of TNF-α and IL-6, together with higher levels of transforming growth factor-β 28 days post-vaccination [190]. Intramuscular co-administration of calcitriol, also known as 1α,25-dihydroxyvitamin D_3_, the active form of vitamin D, at a site adjacent to an influenza vaccination did not enhance subsequent serum hemagglutination inhibition titers to any of the vaccine antigens compared to a placebo [191]. In a recent RCT, oral vitamin D supplementation or simulated sunlight exposure beginning three days after a hepatitis B vaccination, and achieving vitamin D sufficiency within five weeks, did not influence the response to vaccination [192]. Although more studies are needed to build stronger evidence [193], given the potential benefits of vitamin D for immune health in general and the safety of its supplementation, “there is nothing to lose and much to gain by achieving an optimal vitamin D status” in those people affected by COVID-19 [194].

Ultraviolet radiation exerts immunomodulatory effects independent of vitamin D [195]. For instance, ultraviolet radiation-induced immunosuppression is key to the development of carcinogenesis in the skin [196]. In addition to causing immune suppression, exposure to ultraviolet light induces a shift from a Th1- to a Th2-mediated response, increases regulatory T cell function, augments macrophage differentiation, and inhibits plasma cell differentiation [197]. However, the clinical implications of these effects are not entirely clear. On the one hand, the skin area that has been exposed chronically to ultraviolet radiation (such as that above the deltoid muscle) may not be an optimal site for the delivery of vaccines because their efficacy could be compromised, with unexposed sites (e.g., buttock, inside of the upper arm) being potentially more suitable [198]. Moreover, higher levels of antibody titers were found in children who received the rubella vaccine in the winter (with lower exposure to ultraviolet radiation and hence lower vitamin D levels) compared with their summer-inoculated peers [199]. This suggests that sun exposure may impair the efficacy of vaccines. By contrast, hepatitis B vaccine responses have proven to be poorer in winter than summer [192]. In cold and temperate climates, annual epidemics of influenza and the common cold occur during autumn and winter [22], when there is less sunlight and hence lower levels of vitamin D-stimulating ultraviolet radiation. Furthermore, the influenza virus is rapidly inactivated when exposed to ultraviolet radiation from sunlight [200]. Interestingly, both SARS and COVID-19 have emerged during winter months [201] and a recent study estimated that cold and dry weather, together with low levels of ultraviolet radiation, is moderately associated with higher SARS-CoV-2 transmissibility [202]. All this being said, it is too early to draw definitive conclusions on sun exposure and COVID-19.

## 6. Stress

The prevalence of mental health issues increased in April 2020 compared to pre-COVID-19 trends [203,204], with a recent systematic review and meta-analysis reporting that approximately one-third of the general population showed symptoms of stress during the COVID-19 pandemic [205]. Psychological stress can trigger immune dysfunction [206]. Brief episodes of stress, like the ones experienced during an exam or a first date, tend to suppress cellular immunity while preserving humoral immunity, whereas chronic stressors are associated with the suppression of both cellular and humoral measures [207]. Psychological stress has been associated in a dose-response manner with a higher risk of acute respiratory infections [208], and the link between psychological stress and several chronic conditions is well established, particularly for clinical depression, CVD, and human immunodeficiency virus/acquired immune deficiency syndrome [209]. There is meta-analytic evidence for a direct association between acute stress and inflammatory biomarkers (IL-1β, IL-6, IL-10, TNF-α) [210]. Several forms of chronic stress (such as job stress, immigration status, or poverty) have been correlated to elevated levels of hsCRP or CRP [211,212,213]. Conversely, there is recent meta-analytical evidence that mindfulness-based interventions aiming to reduce stress can induce modest but significant reductions in markers of LGCI (hsCRP, IL-6, TNF, and NF- κB activation) [214]. Chronic stress is also believed to trigger ‘inflammaging’ (that is, the chronic LGCI that is frequently associated with aging), partly through increases in oxidative stress [215]. This might also be relevant for vaccine efficacy. Indeed, a meta-analysis concluded that psychological stress could decrease antibody response to influenza vaccination [216]. However, stress levels in the 10 days after influenza vaccination appeared to be more influential to the antibody response than stress in the 2 days prior, with stress-related loss of sleep being primarily responsible for reducing the humoral immune response post-vaccination [217]. Regardless, both short-term (e.g., an academic examination) and long-term stressors (e.g., caregiving) can impair vaccine responses [218,219,220,221,222,223]. A poorer virus-specific T-cell response following influenza vaccination was observed in caregivers of Alzheimer’s disease patients compared to control individuals [224]. The aforementioned evidence suggests that psychological stress can contribute to LGCI and impair the normal response to infections and vaccines, which is relevant to the current pandemic situation.

Although we didn’t find evidence directly linking COVID-19 infection with psychological disorders (such as depression), the stressful situations faced by the overall population during the pandemic are likely to impair immune function and, consequently, increase the risk of SARS-CoV-2 infection and perhaps even affect vaccination efficacy. Accordingly, stress management techniques (e.g., meditation, relaxation techniques and Yoga) that modulate the immune response through various mechanisms (e.g., reducing LGCI, as indicated by lowered levels of circulating IL-6 and TNF-α [214]) could be a potentially effective strategy to attenuate the virus effect on health [214,225,226,227]. There is also meta-analytic evidence linking regular PA with 45% and 28–48% lower odds of depression and anxiety symptoms, respectively [228]. In addition, a recent umbrella review including 16 meta-analyses and 152 RCT concluded that regular physical exercise can be an effective adjunctive treatment for improving symptoms across a broad range of mental disorders (such as anxiety, depression, and post-traumatic stress disorder) [229].

## 7. Sleep and Circadian Disruption

Numerous behaviors that are prevalent in westernised countries can result in sleep disorders, including shift work, long working hours, as well as 24-h access to artificial light (shops, telephone, television, or the Internet). In this context, sleep disturbances are emerging as another consequence of the COVID-19 pandemic. There is longitudinal evidence that the lockdown, imposed during the pandemic, had a negative impact on the sleep quality of a Spanish cohort [27]. Moreover, a study in over 5000 Canadian adults showed that the proportion of individuals with clinically meaningful sleep difficulties markedly increased from before (36%) to after the COVID-19 pandemic (50.5%) [230].

Lack of sleep and circadian disruption is associated with immune dysfunction and a pro-inflammatory status [13,231,232]. Sleep deprivation upregulates inflammatory cytokines such as TNF-α, IL-1, or IL-6 [233]. In older adults, ageing-related alterations in nocturnal wake time and daytime sleepiness are associated with elevations of both plasma IL-6 and cortisol concentrations [234]. In fact, Atienza et al. suggested that the combination of abnormal sleep, circadian disruption, and impaired immune response promotes inflammation [235]. In addition, sleep deprivation is associated with the decline in the number of myeloid dendritic cell precursors producing IL-12, a main inducer of Th1 responses [236]. These results support the importance of an optimal sleep for health maintenance, particularly in the context of the current COVID-19 pandemic. On the other hand, a recent meta-analysis found that long sleep duration, as well as sleep disturbances, was associated with higher levels of CRP and IL-6 [237]. A study with 1,310 individuals from Italy showed that, during home confinement, participants reported a lower sleep quality despite spending more time in bed [238]. This finding might be attributed to various factors such as stress and circadian disruption. The latter can be caused by exposure to light at night, which is associated with reduced melatonin synthesis [239,240]. Melatonin has important properties as an immunomodulator agent that exerts anti-inflammatory effects [241,242] while also stimulating the production of NK and CD4+ cells and inhibiting the release of CD8+ cells [243]. Yet the most documented function of melatonin is the regulation of circadian rhythms. In this regard, circadian disruption (independently of sleep loss), such as that suffered by night-shift workers, has been shown to increase hsCRP levels [244,245].

Since inadequate sleep along with circadian disruption can promote LGCI and contribute to inmunosenescence, it might also affect the immune response to infections and vaccines. In fact, each additional hour of sleep was shown to increase the secondary antibody levels after hepatitis B vaccination by 56% [246], whereas sleeping <6 h per night was associated with a lower likelihood of showing a clinically protective response to the vaccination vs. sleeping <7 h per night [246]. Compared to no sleep, sleep enhances immune memory, thereby generating benefits of antigen-specific T-helper cell response after hepatitis A vaccination that were maintained one year later [247]. On the other hand, more studies are needed to confirm whether acute sleep deprivation can affect the human antibody titer response to vaccination, since short-term studies have yielded conflicting results. For instance, sleep restriction before and after influenza vaccination, despite a prolonged period of sleep recovery following vaccine administration, decreased antibody titers 10 days after vaccination [248]. However, the short-term negative effects of sleep on the antibody response apparently disappeared from 3 to 4 weeks after vaccination, because antibody titers no longer differed among sleep-restricted individuals and those who maintained their usual bedtime prior to receiving the vaccine [248]. Likewise, sleep deprivation in the night after vaccination against influenza virus caused a lower antibody titer response 5 days after vaccination, although it did not affect antibody titers thereafter [249]. Notwithstanding, evidence consistently suggests a key a role for circadian rhythms and sleep on immune system homeostasis [250] and hence the timing of vaccination might also affect the immune response. Accordingly, men vaccinated in the morning vs. afternoon had a better peak antibody response to both hepatitis A and influenza vaccines [251].

Given the importance of sleep for immune health and the negative effect of the COVID-19 pandemic on sleep characteristics in most individuals, the promotion of ‘chronic’ interventions aimed at improving sleep quality such as regular exercise [252] and circadian synchronization [253] should be kept in mind in view of future pandemics.

## 8. Exposure to Environmental Pollution

Exposure to persistent organic pollutants—that is, chemical substances that have a long half-life in the environment and can be harmful to human health and/or the environment—could further aggravate the impact of pandemics. Air pollution has an adverse effect on global disease burden, and it has been identified as the fifth-largest mortality risk factor worldwide, representing 7.6% of all-cause mortality [254]. The use of pesticides and agricultural or industrial chemicals together with exposure to hazardous waste (e.g., electronic waste) is increasing, together with the spectrum of adverse effects on human health [255,256]. A special situation exists for the so-called *xenobiotics*. These are chemical substances found within an organism that are not naturally produced (or expected to be present) within the organism in question. An example is bisphenol A, a ubiquitous plasticizing agent found in food, beverage cans, and thermal receipt paper. Xenobiotics represent a stress factor for immune cells and can cause inflammation [257]. Tobacco smoking, which will be discussed in a later section, is another source of xenobiotics that has been associated with a variety of deleterious effects on human health [258].

Long-term exposure to air pollution leads to the overexpression of ACE2, thereby facilitating viral penetration and subsequent depletion of ACE2 and increasing the likelihood of poor outcomes of COVID-19 [259]. Exposure to air pollution may also influence the systemic inflammatory response [260], the alveolar macrophage-mediated inflammatory response to phagocytize the virus [261], and affect host immunity [262]. There is meta-analytical evidence suggesting that exposure to ambient pollutants is associated with an increased level of CRP. On the other hand, ambient pollutants can increase Th2 immune responses, which is a characteristic in the respiratory tract during severe virus-induced exacerbations [263], such as asthma and chronic obstructive pulmonary disease. The decreased immune response caused by air pollution could also affect vaccination efficacy. Exposure to metals such as mercury has been associated with a lower immune response to vaccination programs including hepatitis B, influenza, measles, pertussis, tetanus, and diphtheria in children [138]. However, further studies are needed to corroborate this association.

The bulk of evidence suggests that exposure to air pollution might increase the risk of respiratory infections and, consequently, contribute to a worse prognosis of COVID-19. While the COVID-19 pandemic has resulted in a large drop in pollution levels [264], exposure to air pollution may increase the odds of COVID-19 infection [259,265] as well as symptoms severity [259,266] and risk of death [259,267,268]. In terms of COVID-19 mortality, of the 4443 fatality cases recorded in 66 administrative regions from Italy, Spain, France, and Germany, a vast majority (83%) of COVID-19 fatalities occurred in the regions with the highest nitrogen dioxide levels [268]. Coronavirus is in the air [269], and ultraviolet reduction as a consequence of air pollution may promote viral persistence in the air [270]. Exposure to toxic metals such as arsenic, lead, cadmium, or mercury is associated with respiratory dysfunction and respiratory diseases (i.e., chronic obstructive pulmonary disease and bronchitis), and hence a link between metal exposure and COVID-19 risk and/or severity might exist [271].

Therefore, it seems therefore reasonable to promote interventions aimed at reducing pollution levels.

## 9. Smoking

Approximately 1.3 billion people smoke worldwide [272]. Despite the anti-smoking public health policies implemented over the last 50 years, tobacco smoking remains a leading global risk factor [258]. Smoking history decreases life expectancy by at least a decade compared to those who have never smoked [273]. In fact, the smoke we inhale from tobacco contains more than 60 carcinogens [274].

There is accumulating evidence that like SARS-CoV, SARS-CoV-2 utilizes the cell membrane receptor ACE2 [275] as the main entry point into target cells. One of the main constituents of cigarette smoke, nicotine, might be able to downregulate the expression or activity of ACE2 receptors [276]. It could be thus hypothesized that inhaled nicotine during smoking has a protective effect against COVID-19. A recently published report, however, found that pulmonary ACE2 receptors are more activated in smokers than in never smokers [277], indicating that smoking is potentially a risk factor for COVID-19 by modulating ACE2 expression.

Smoking is also a potential risk factor for COVID-19 because of its detrimental effects on lung function and the possible transmission of SARS-CoV-2 by finger–mouth contact during tobacco use. However, initially, there was controversy regarding the relationship between smoking and SARS-CoV-2 infection/disease severity [278,279,280,281], and there were conflicting hypotheses on the role of nicotine on immune function [12]. Some studies have suggested that nicotine has anti-inflammatory effects [282,283] that could be beneficial in attenuating the cytokine storm often produced in response to viral infection [281,284,285]. There is indeed some evidence supporting a therapeutic role for nicotine in patients with severe COVID-19 [284,285], which is consistent, at least partly, with the notion that, to some extent, COVID-19 might be a disease of the nicotinic cholinergic system [281]. However, meta-analytical evidence indicates that smoking worsens the prognosis of COVID-19 [286,287,288,289].

It should also be considered that cigarette smoking and vaping can weaken immune health and increase the risk of infection and outcomes [290,291,292], potentially making smokers more vulnerable to SARS-CoV-2 and future viruses. For instance, influenza risk is several-fold higher (and the clinical presentation of the disease is much more severe) in smokers than non-smokers [291]. On the other hand, the number and activity of NK cells are decreased in smokers [293,294,295]. In addition, higher production of the proinflammatory cytokines IL-1β, IL-6, and TNF-α together with an enhanced proliferative response to mitogens has been reported in smokers compared to non-smokers [295]. The molecular pathways by which tobacco exerts deleterious effects on the immune system would involve NFκB, mitogen-activated protein (commonly known as ‘MAP’) kinases signaling as well as histone modification [296]. Of note, the high level of toxic metals in tobacco smoke may also partly underlie the association between smoking and COVID-19 severity due to their role in the development of respiratory dysfunction, immunotoxicity, and severity of viral diseases [271].

There is meta-analytical evidence that smokers have a 1.53 higher risk of nonresponse to vaccines such as hepatitis B compared to non-smokers [71]. This impaired response to vaccination might be mediated by the proinflammatory status associated with smoking [297]. Another potential mechanism has been postulated in a study with a murine model showing that the impaired response to vaccines may be mediated by cigarette smoke-related inhibition of the pulmonary T-cell response to vaccination against influenza virus and *Mycobacterium tuberculosis* [298].

Further research is warranted when keeping in mind that there is also preclinical evidence suggesting that smoking could be protective against the infection and severity of COVID-19 [278,279,280,281]. However, in light of the multiple well-established adverse effects of smoking on human health and the lack of evidence on whether an eventual association between smoking and protection against COVID-19 reflects an actual causal effect, public health programs should continue to support smoking cessation.

## 10. Gut Microbiome

The gastrointestinal tract is inhabited by about 100 trillion microbes (or *microbiota*) [299], including mainly bacteria, collectively known as the *microbiome*, that regulate fundamental functions that preserve human health, including host nutrient metabolism, xenobiotic and drug metabolism, or maintenance of structural integrity of the gut mucosal barrier^2^. The gut microbiome also plays a pivotal role in immune tolerance, inflammation, and protection against pathogens [300]. In fact, the microbiome has an important effect on the training and development of major components of the host’s innate and adaptive immune system [300]. Alterations of the gut microbiome (known as dysbiosis) might lead to dysregulated immune responses to commensal microbes and the stabilization of a proinflammatory community of microbes, hence contributing to LGCI [301]. Accordingly, dysbiosis is associated with a wide variety of conditions including obesity [302], type 2 diabetes [302], hypertension [303], colon cancer [304], autoimmune diseases (such as inflammatory bowel disease [305]), allergic asthma [306], and human immunodeficiency virus [307].

Although the gut microbiome is a part of the exposome, several of the aforementioned exposome factors can influence the status of the gut microbiome and lead to dysbiosis [108,308], reflecting the bidirectional and dynamic relationship between different components of the exposome, as well as the host. The inappropriate use of antibiotics and other medications (e.g., proton pump inhibitors, antipsychotics, opioids, and nonsteroidal anti-inflammatory drugs) has a notable impact on the overall architecture of the gut microbiome [309,310]. Other factors shown to influence the composition and function of the gut microbiome include physical exercise [311], psychosocial stress [312], tobacco and alcohol use [313,314], and, as mentioned before, diet [107,108]. For instance, a high-sodium intake can alter the composition of the gut microbiome [96], and this has been associated with increased and decreased pro- and anti-inflammatory activity, respectively, of CD4+ T cells and macrophages [96]. Likewise, a high-fat/low-fiber westernised diet is linked to a decrease in microbial diversity and species richness, with a low abundance of some beneficial species (such as *Bifidobacterium*, *Lactobacillium* or *Eubacterium*), whereas a Mediterranean diet has essentially the opposite effect [315]. On the other hand, probiotics, live microorganisms with purported health benefits for the host if consumed in adequate amounts, and microbiota-accessible carbohydrates (commonly abbreviated by ‘MACs’ and also known as prebiotics—carbohydrates that are resistant to digestion and are made available for gut microbiota to ferment or metabolize into beneficial compounds, such as short chain fatty acids) could favourably modulate the gut microbiota, thereby exerting immunomodulatory and anti-inflammatory effects [107,108,316,317]. Accordingly, low-fat yogurt, a fermented dairy product containing a variety of probiotic bacteria, is associated with a reduction in markers of chronic inflammation and abdominal obesity in interventional and observational studies [318,319,320]. On the other hand, paradoxically, the hygiene measures proposed to prevent COVID-19, such as hand washing, could alter the composition of the gut microbiome. According to the ‘hygiene hypothesis’, which proposes that exposure to germs and certain infections helps the development of the immune system, excessive hygiene measures could negatively affect the microbiome [321].

Therefore, based on the influence of the microbiome on immunity, and the existence of a crosstalk between the gut microbiota and the lungs, known as the gut–lung axis [322,323], it might be argued that a healthy gut microbiome could play an important role in preventing respiratory infections or at least attenuating their severity. Indeed, the microbiota has previously been shown to regulate immune defence against influenza virus infection in the respiratory tract [324]. More recently, some authors have suggested the existence of dysbiosis in COVID-19 patients [325,326]. Zuo et al. [325] found an inverse correlation between the abundance of the gut species *Faecalibacterium prausnitzii* (an anti-inflammatory bacterium) and disease severity. Yeoh et al. concluded that the gut microbiome is implicated in the magnitude of COVID-19 severity, possibly through the modulation of host immune responses [327]. These authors also found that even 30 days after disease resolution, gut microbiome composition was still altered, which could contribute to persistent symptoms [327]. In addition, there is evidence for an increased incidence of dysbiosis in critically ill patients, a phenomenon associated with sepsis, organ failure, and death [328,329].

A recent systematic network and meta-analysis analysed the potential antiviral mechanisms of probiotics [330]. The authors found that probiotics could affect ACE2-mediated virus entry and temper the pro-inflammatory status caused by activation of nucleotide-binding oligomerization domain (NOD), leucine-rich repeat (LRR)-containing proteins (NLR) P3 (NLRP3) inflammasome, with *NLRP3* inflammasome being a multimeric protein complex that initiates an inflammatory form of cell death and triggers the release of proinflammatory cytokines IL-1β and IL-18 [331]. Probiotics can also improve the systemic immune response to viral infection (thereby attenuating the resulting lung tissue damage and cardiovascular complications) and modulate glucose/lipid metabolic pathways affected by the infection [330]. These findings might suggest that probiotics could be considered a potential preventive and alternative treatment strategy for both mild and severe stages of COVID-19 [330], although the evidence is still limited.

The gut microbiome could also affect the immune response to vaccination [332,333]. There is reasonable evidence that the gut microbiome improves both B cell and T cell responses to vaccination [334]. Of note, vaccine responses can vary widely between people in a given region [335]. A possible explanation is the high variability in the types of gut microbiota between populations [334]. For example, a study conducted in Ghanaian children concluded that the gut microbiome composition, which correlates significantly with rotavirus vaccine immunogenicity, might contribute to the diminished efficacy of rotavirus vaccines reported in developing countries [336]. Another study analysed the influence of gut microbiome on mucosal IgA antibody response to the polio vaccine in a population of Chinese infants [337]. The authors found that the composition of the gut microbiome was significantly different, reporting a higher and lower abundance of *Firmicutes* and *Actinobacteria*, respectively, in IgA-negative children than in their IgA-positive peers [337]. On the other hand, antibiotic administration in individuals with low levels of pre-existing immunity impairs responses to a seasonal influenza vaccine [338]. Conversely, a recent systematic review on the role of probiotics on vaccine responses suggested that probiotics represent a relatively cheap intervention to improve vaccine efficacy and duration of protection [339].

Keeping a healthy gut microbiome represents an important first-line defence against pathogens such as SARS-CoV-2, regardless of their virulence. As for the potential impact of the gut microbiome in modulating vaccine immunogenicity, further work in different human populations is needed.

## 11. Limitations and Perspectives

This review has some methodological limitations that should be considered. First, its narrative nature is likely to induce some bias, as it lacks strict criteria for the inclusion/exclusion of studies. In addition, the overwhelming number of new studies published virtually every day on the COVID-19 pandemic implies that previous conclusions on a given area are frequently challenged and must be revisited, with new hypotheses frequently arising. In this regard, we finished writing the first draft of this review on 17 July 2021, and therefore, at the time this review is published, evidence might have been updated on certain topics. On the other hand, although exposome improvements seem to be a potentially effective strategy to deal with COVID-19, a vast amount of research still needs to be implemented not only to shed light on the effects of combining a healthy lifestyle with environmental exposure but also to disentangle potential pathophysiological underpinnings. After reviewing the current literature, we have provided practical, testable hypotheses for future research in the field.

## 12. Conclusions

**Figure 3:**
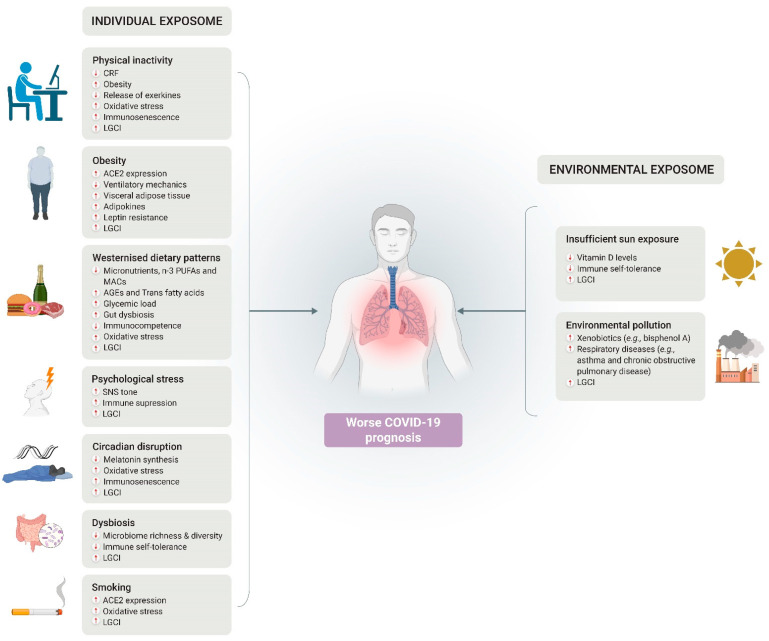
Summary of potential mechanisms underlying the negative effects of the different exposome components on the prognosis of COVID-19. Abbreviations: ACE2, angiotensin converting enzyme-2; AGEs, advanced glycation end products; CRF, cardiorespiratory fitness; LGCI, low-grade chronic inflammation; MACs, microbiota-accessible carbohydrates; PUFA, polyunsaturated fatty acids; SNS, sympathetic nervous system. Source: Self-elaboration based on the main results obtained in the scientific literature.

Throughout the COVID-19 pandemic, a variety of lifestyle and environmental exposures, collectively referred to as the exposome, that are known to play a major role in immune health, have been worsened. Notably, these include an increased prevalence of physical inactivity and obesity, unhealthy dietary patterns, high levels of psychosocial stress, sleep deprivation and circadian disruption, as well as high exposure to air pollution, low sun exposure, and insufficient vitamin D levels (Figure 3). The need to implement ‘traditional’ measures aimed at avoiding viral transmission (e.g., home confinement, closure of parks and gyms) should not overshadow the deleterious effects that they can have on other health markers. As a society, we should be prepared for a potential recurrence of previous pandemias and the emergence of new ones, and this preparedness should start with the promotion of healthy lifestyles and environmental exposures.
